# Direct targets of Klf5 transcription factor contribute to the maintenance of mouse embryonic stem cell undifferentiated state

**DOI:** 10.1186/1741-7007-8-128

**Published:** 2010-09-27

**Authors:** Silvia Parisi, Luca Cozzuto, Carolina Tarantino, Fabiana Passaro, Simona Ciriello, Luigi Aloia, Dario Antonini, Vincenzo De Simone, Lucio Pastore, Tommaso Russo

**Affiliations:** 1 CEINGE Biotecnologie Avanzate, Via Gaetano Salvatore 482, 80145 Naples, Italy; 2 European School of Molecular Medicine (SEMM), Via Gaetano Salvatore 482, 80145 Naples, Italy; 3 Dipartimento di Biochimica e Biotecnologie Mediche, Università di Napoli "Federico II", Via Sergio Pansini 5, 80131 Naples, Italy

## Abstract

**Background:**

A growing body of evidence has shown that Krüppel-like transcription factors play a crucial role in maintaining embryonic stem cell (ESC) pluripotency and in governing ESC fate decisions. Krüppel-like factor 5 (Klf5) appears to play a critical role in these processes, but detailed knowledge of the molecular mechanisms of this function is still not completely addressed.

**Results:**

By combining genome-wide chromatin immunoprecipitation and microarray analysis, we have identified 161 putative primary targets of Klf5 in ESCs. We address three main points: (1) the relevance of the pathways governed by Klf5, demonstrating that suppression or constitutive expression of single Klf5 targets robustly affect the ESC undifferentiated phenotype; (2) the specificity of Klf5 compared to factors belonging to the same family, demonstrating that many Klf5 targets are not regulated by Klf2 and Klf4; and (3) the specificity of Klf5 function in ESCs, demonstrated by the significant differences between Klf5 targets in ESCs compared to adult cells, such as keratinocytes.

**Conclusions:**

Taken together, these results, through the definition of a detailed list of Klf5 transcriptional targets in mouse ESCs, support the important and specific functional role of Klf5 in the maintenance of the undifferentiated ESC phenotype.

See: http://www.biomedcental.com/1741-7007/8/125

## Background

Embryonic stem cells (ESCs) are derived from the inner cell mass of preimplantation embryos; they are amenable to manipulation, enrichment and expansion and retain the developmental potency of embryonic founder cells, being able to differentiate into cells and tissues of all three germ layers both *in vitro *and *in vivo *[1,2]. For these characteristics, ESCs represent an invaluable source of different cell types, thus opening up new possibilities for cell therapy. The understanding of the transcriptional regulatory networks that operate in ESCs is fundamental to unravel the molecular basis of pluripotency, self-renewal and reprogramming. The complexity of this regulatory network was highlighted by the identification of hundreds of genes, targets of the ESC master genes Oct3/4, Nanog and Sox2 [3,4]. In addition to these master regulatory factors, several other transcription factors play important roles in the control of this regulatory network [5-9], such as Krüppel-like factors (Klfs).

Klfs belong to the Sp1 family of transcription factors with over 20 members [10,11]. Three members of this family (Klf2, Klf4 and Klf5) are expressed in undifferentiated mouse ESCs and downregulated during early stages of differentiation [12]. Klf4 is one of the original "Yamanaka factors" required to reprogram somatic cells to a pluripotent state [13], and Klf2 and Klf5 are able to substitute for Klf4 function in the reprogramming, including cocktail [14]. It has been proposed that Klf2, Klf4 and Klf5 have redundant functions because the impairment in the ESC undifferentiated state was only observed following knockdown (KD) of all these three genes [15]. Nevertheless, there is evidence that Klf5 has unique functions: We have recently shown that KD of Klf5 induces ESC differentiation, whereas its ectopic expression is able to maintain ESC pluripotency in absence of leukemia inhibitory factor (LIF) [16] and Klf5-knockout (KO) mice show developmental defects at the blastocyst stage [17], thus suggesting a specific requirement of this Klf in early embryogenesis and in turn a high hierarchical role in the transcriptional network. Opposite to other transcription factors regulating ESC pluripotency and the first step of embryo development, Klfs are also expressed in adult terminally differentiated cells [18]. Klf5 is expressed in skin, intestinal crypts, stomach, lung, testis, uterus, heart and kidney [19-21]. We systematically explored Klf5-specific targets in mouse ESCs by matching gene expression profiling with chromatin immunoprecipitation coupled with parallel short tag-based sequencing (ChIP-seq) that identifies all Klf5 genome binding sites. Here we report that Klf5 regulates at least 313 genes functioning as a repressor or activator. A total of 161 of 313 genes bear at least one binding site for Klf5 within 100 kb from the gene boundaries, indicating that they can be considered Klf5 primary targets. We first demonstrated that suppression or ectopic expression of some Klf5 targets strongly affects the ESC undifferentiated state, thus further supporting a high hierarchical role of this transcription factor in ESCs. Then we addressed the specificity of Klf5 function in ESCs by demonstrating that Klf2 and Klf4 have nonredundant roles and that Klf5 targets in ESCs are different from the gene targets of this transcription factor in adult differentiated cells.

## Results

### Identification of Klf5 direct targets

**Figure 1:**
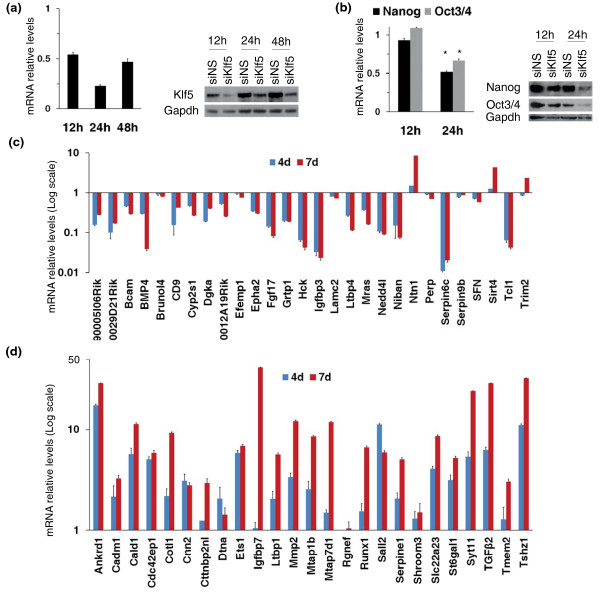
**Identification of Klf5 targets by microarray analysis**. **(a) **qPCR and western blot analysis of Klf5 expression following transfection of siKlf5 and siNS at different time points (12, 24 and 48 hr after siRNA transfection). **(b) **Expression level of stemness markers was measured 12 and 24 hr after transfection by qPCR and Western blot analysis. All qPCR data are expressed as fold changes relative to mock-transfected cells. Error bars represent standard errors (SD) of triplicates. *P < 0.01. **(c **and **d) **Expression of 53 Klf5-target genes measured by real-time PCR in undifferentiated and 4-day (4 d) and 7-day (7 d) differentiated ESCs. Both Klf5-induced genes **(c) **and Klf5-repressed genes **(d) **are reported. The results are reported as fold changes relative to undifferentiated cells.

To identify genes directly regulated by Klf5, we first analyzed the transcriptome changes upon Klf5 KD in ESCs by microarray analysis. To minimize indirect effects due to phenotypic changes induced by Klf5 KD, we performed a time course analysis both by qPCR and by Western blot analysis, which showed that Klf5 expression is significantly decreased already 12 hr after siRNA transfection (Figure 1a). On the contrary, the expression of stemness genes such as Oct3/4 and Nanog, previously demonstrated to be modified by Klf5 KD [16], is still unaffected at 12 hr (Figure 1b). Therefore, it is possible to capture early effects on transcriptome following Klf5 KD at 12 hr after transfection.

Statistically significant probes (FDR < 0.1) were classified as upregulated and downregulated following Klf5 KD by using as cut-off > 1.25- and < 0.75-fold changes, respectively. On the basis of these constraints, we identified 239 upregulated and 74 downregulated genes (Additional file 1).

These predicted genes encode a set of proteins involved mainly in development and differentiation (Additional file 2), in agreement with the Klf5 role in ESCs and embryonic development [16,17]. Interestingly, 52 genes controlled by Klf5 encode regulators of transcription, indicating that Klf5 regulates many transcription factors, which in turn regulate their downstream targets. Moreover, among the genes regulated by Klf5, there are factors that have already demonstrated a role in ESC pluripotency, such as Tcl1 [8,22], BMP4 [23], Nr0b1 [24] and CD9 [25]. We also compared our results with available data [15,17,26,27], and we found that 86 of 96 transcripts showed the same behavior upon Klf5 KD and upon triple KD of Klf2, Klf4 and Klf5 [15] (Additional file 3). Moreover, we found that 75% of the transcripts showed the same trend in our analysis and in Klf4 KD cells [26] (Additional file 3). Instead, no significant correlation was found when our data were compared with the data of LIF deprivation or expression of STAT3 dominant-negative form [27] (Additional file 3).

It is reasonable to assume that Klf5-regulated genes, identified by gene expression profiling, include both direct and indirect transcriptional targets. To distinguish between direct target genes and those indirectly regulated through alterations in transcriptional networks governed by Klf5, we employed genome-wide chromatin immunoprecipitation (ChIP). We introduced a FLAG-tagged Klf5 in ESCs and generated two independent pools of stable clones. The levels of Klf5 were assessed by Western blot analysis with anti-Klf5 and anti-FLAG antibody to detect both endogenous and exogenous Klf5 (Additional file 4). Chromatin from these cells was immunoprecipitated with anti-FLAG antibody, and purified DNA fragments were analyzed by direct high-throughput sequencing. We identified 6480 putative binding sites, and among these 5820 had an FDR lower than 5% (Additional file 5). ChIP-qPCR validations were carried out on 15 Klf5-binding sites with different numbers of ChIP tag counts within the defined overlap region (Additional file 4). Twelve of 15 target loci subjected to validation showed a significant enrichment over three independent control regions (Additional file 4).

Next, we analyzed Klf5-ChIP target sequences with the motif-finding CisFinder software to search for over-represented motifs. The three best results were represented by CG-rich elements (Additional file 6), in agreement with previous reports [28]. Moreover, all three motifs contained the CTGC sequence, suggesting a putative binding site for Klf5.

To identify primary targets of Klf5 among the genes regulated upon Klf5 KD, we matched microarray and ChIP-seq data, and we found that the relationship between target promoter occupancy and gene expression analysis is excellent. In fact, we found 161 of 313 genes whose expression is regulated by Klf5, showing a Klf5 binding site within 100 kb from the gene boundaries (Table 1, Additional file 5). To validate microarray data among these 161 Klf5 putative primary targets, we selected 60 genes where Klf5 binding sites are at various distances from gene boundaries. qPCR analysis of these genes performed upon Klf5 KD revealed that 53 (>88%) of 60 showed the same trend observed in microarray analysis (Additional file 7).

**Table 1:** List of Klf5-target genes obtained by matching microarray and ChIP-seq data. Klf5-repressed and activated genes bearing a binding site for Klf5 within 100 kb from gene boundaries are reported.

Klf5-repressed target genes				Klf5-activated target genes	
1110007C09Rik	Col5a1	Irx3	Pou3f1	1190005I06Rik	F5
1500005I02Rik	Cotl1	Itga7	Rasl11b	1200015N20Rik	Fgf17
2310045A20Rik	Ctgf	Itpr1	Rgnef	1600029D21Rik	Gjb5
Adam12	Cttnbp2nl	Jak2	Runx1	8430410A17Rik	Grtp1
Adam19	Dbndd2	Kif21a	Sall2	AA409316	Hck
Ankrd1	Dll1	Kif5c	Sema3e	Abcb1b	Igfbp3
Anxa5	Dnmt3a	Limd2	Serpine1	Adora1	Klk1b21
Atf3	Dtna	Lrrk1	Serpine2	Ap1m2	Krt17
Auts2	Efnb2	Lrrn1	Shroom3	Apobec2	Lamc2
AW548124	Elk3	Ltbp1	Slc22a23	Ass1	Ltbp4
Bach1	Errfi1	Maff	Specc1	Bbs2	Mras
Bcar1	Ets1	Mcam	Spnb2	Bcam	Nedd4l
Bin1	Fads1	Mfhas1	Spop	Bmp4	Ngfr
Btg1	Farp1	Mmd	St6gal1	Brunol4	Niban
Btg2	Fez2	Mmp2	Syt11	Cd9	Ntn1
C79267	Flt1	Mtap1b	Tax1bp3	Cyp2s1	Perp
Cadm1	Foxa1	Mtap7d1	Tgfb1i1	Depdc6	Pura
Cald1	Fzd2	Mycl1	Tgfb2	Dgka	Rnase4
Cap2	Gadd45g	Myo1b	Tmem2	E130012A19Rik	Serpinb6c
Ccng2	Gap43	Nfil3	Tmem98	Efemp1	Serpinb9b
Cd248	Gata2	Oaz2	Tpm2	Ehmt2	Sfn
Cd40	Gdnf	Otx2	Tshz1	Emp1	Sirt4
Cdc42ep1	Glipr1	Pcdh8	Ugcg	Eno3	Tcl1
Cgnl1	Gpsm1	Pcsk2	Wisp1	Epha2	Trim2
Chd7	Hmga2	Pdgfb	Yaf2		
Cnn1	Hoxb2	Plcg2	Zyx		
Cnn2	Igfbp5	Plekhg2			
Col1a1	Igfbp7	Pls3			
Col1a2	Insm1	Pmp22			

To gain insights into the primary targets of Klf5, we analyzed the expression pattern of the 53 validated genes (Figures 1c and 1d), during neural differentiation of ESCs. Klf5, such as the other two ESC-specific Klfs, is highly expressed in undifferentiated ESCs, and its expression dramatically decreases when differentiation occurs (Additional file 2). We reasoned that genes activated by Klf5 are expected to show the same expression profile of Klf5, i.e., expressed in undifferentiated ESCs and downregulated when differentiation occurs. On the contrary, the genes that are negatively controlled by Klf5 are expected to show an opposite expression profile, i.e., not expressed in undifferentiated ESCs and upregulated when differentiation occurs. As expected, almost all the genes activated by Klf5 are expressed in ESCs and repressed during differentiation (Figure 1). On the other hand, almost all the genes that are inactive or repressed in ESCs, but are expressed during differentiation, appear to be negatively regulated by Klf5 (Figure 1d).

### Suppression of Klf5 target genes impairs ESC undifferentiated state

**Figure 2:**
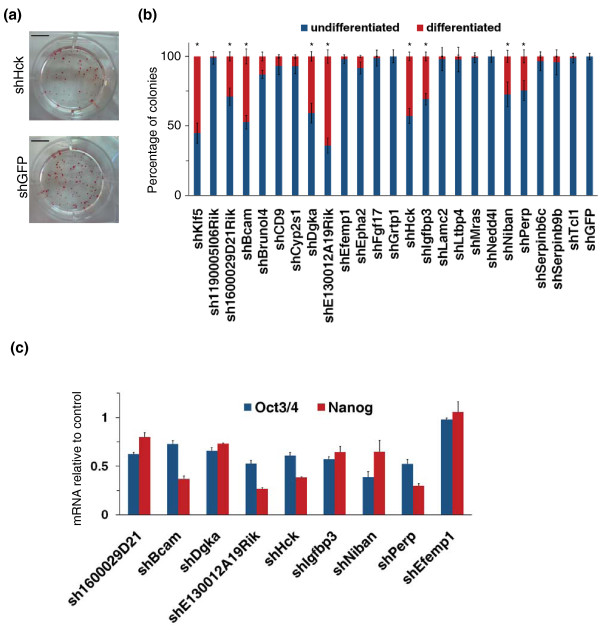
**Effects of KD of Klf5-target genes in ESCs**. **(a) **ESCs stably transfected with shRNA plasmids were grown for 7 days at clonal density (50-100 cells/cm^2^) and then stained for alkaline phosphatase (AP) activity. The results of shGFP-transfected cells (negative control) and shHck-transfected cells are shown as examples. Scale bars, 1 cm. **(b) **Histogram showing the percentages of undifferentiated (blue) and differentiated (red) colonies observed by AP staining after knockdown of Klf5 response genes. shKlf5-transfected cells are the positive control, and shGFP-transfected cells are the negative control. Bars represent SD of triplicates. *P < 0.01. **(c) **qPCR analysis of stemness markers after KD of Klf5 target genes that induced a significant decrease in AP-positive colonies. Efemp1 KD cells were used as negative controls. The data are represented as fold changes relative to shGFP-transfected cells. Error bars represent SD of triplicates. Differences are significant with *P *< 0.01.

The first point we asked is that of the relevance of Klf5 direct targets in the maintenance of ESC phenotype. Among the putative Klf5 direct targets, we decided to start by analyzing 23 genes that were selected on the basis of validation by qPCR (Additional file 7) and their expression profile during ESC differentiation (Figure 1). To this aim, we selected pools of stably transfected ESC clones, where the expression of these 23 genes was knocked down by specific shRNAs. A reduction of at least 20% in transcription levels was observed after selection (Additional file 8). The stably transfected cells were plated at clonal density and further grown for 7 days. The resulting colonies were stained for alkaline phosphatase (AP) activity (Figure 2a) and the percentage of undifferentiated and differentiated colonies was calculated. KD of Klf5 (positive control) resulted in at least 40% decrease of AP-positive colonies compared to control shRNA transfected cells (Figure 2b). Knockdown of 8 of 23 target genes analyzed resulted in a significant decrease of AP-positive colonies (Figure 2b). To confirm that these decreases in the number of AP-positive colonies were due to an impairment of ESC undifferentiated state, we measured the level of stemness markers in cells KD for these eight genes soon after selection. Expression of both Oct3/4 and Nanog was significantly reduced compared to control after knockdown of all these genes (Figure 2c). The observed decrease in Oct3/4 and Nanog expression corresponded to a significant increase of early differentiation markers (Additional file 9). These results were confirmed by further independent shRNAs (Additional file 8). Taken together, these results suggest that Klf5 controls the expression of at least 8 of 23 genes required to maintain the undifferentiated state of ESCs.

### Ectopic expression of Serpine1 impairs ESC undifferentiated state

**Figure 3:**
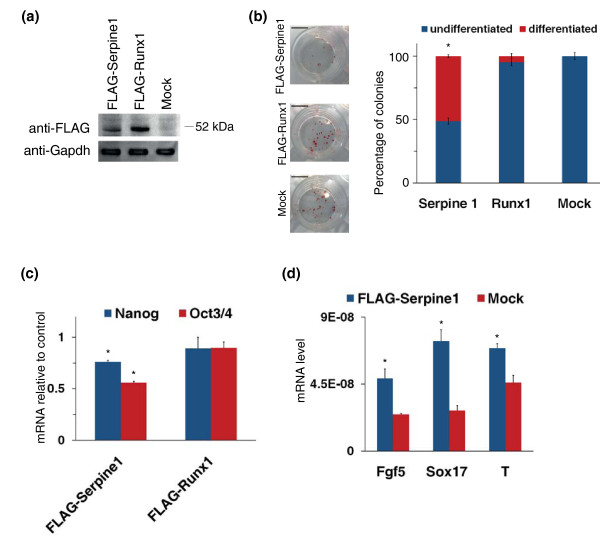
**Ectopic expression of Serpine1 impairs ESC undifferentiated state**. **(a) **ESCs were transfected with empty vector (mock) or FLAG-tagged Runx1 and Serpine1 expression vector. Western blot stained with anti-FLAG antibody show the expression level of FLAG-Serpine1 and FLAG-Runx1 24 hr after transfection. **(b) **ESCs transfected with empty vector, FLAG-Serpine1 or FLAG-Runx1 were grown at clonal density (50-100 cells/cm^2^) for 7 days and then stained for alkaline phosphatase activity (scale bars, 1 cm). The percentage of undifferentiated (blue) and differentiated (red) colonies is reported. Bars represent SD of triplicates. *P < 0.05. **(c) **Expression level of stemness markers was measured 48 hr after transfection by qPCR. The data are expressed as fold changes relative to mock-transfected cells. Error bars represent SD of triplicates. *P < 0.01. **(d) **Expression levels of early differentiation markers of ectoderm (Fgf5), endoderm (Sox17) and mesoderm (T, also known as brachyury) were measured 48 hr after transfection by qPCR. Error bars represent SD of triplicates. *P < 0.01.

As shown above, we found many genes whose expression in ESCs seems to be negatively regulated by Klf5. Two of them, Serpine1 and Runx1, were selected for functional studies. Their Klf5-dependent regulation is confirmed by the observation that they are expressed at very low levels in undifferentiated ESCs. Furthermore, these genes that seem to be negatively regulated by Klf5 in undifferentiated ESCs showed a strong induction upon ESC differentiation when Klf5 disappeared (Figure 1d) [16]. Thus, we investigated their effect on stemness by forced expression in undifferentiated ESCs. We transfected ESCs with expression vectors bearing FLAG-tagged Serpine1 or Runx1 (Figure 3a); these cells, grown at clonal density for 7 days, were stained to assay AP activity. Alkaline phosphatase staining showed that forced expression of FLAG-Serpine1 is able to induce a significant decrease in the number of undifferentiated colonies, whereas FLAG-Runx1-transfected cells did not show a significant decrease in AP expression (Figure 3b). To further study the effect of forced expression of Serpine1 and Runx1, we measured the expression level of Oct3/4 and Nanog. As shown in Figure 3c, the expression of Oct3/4 was impaired upon Serpine1 constitutive expression in ESCs, and also Nanog showed a moderate but significant decrease, whereas no significant changes were detectable in FLAG-Runx1-expressing cells. This decrease of Oct3/4 and Nanog levels corresponded to a significant increase of early differentiation markers (Figure 3d) even in the presence of LIF, indicating that Serpine1 alone is able to impair the undifferentiated state by inducing an uncontrolled differentiation.

### ESC-specific targets of Klf5

**Figure 4:**
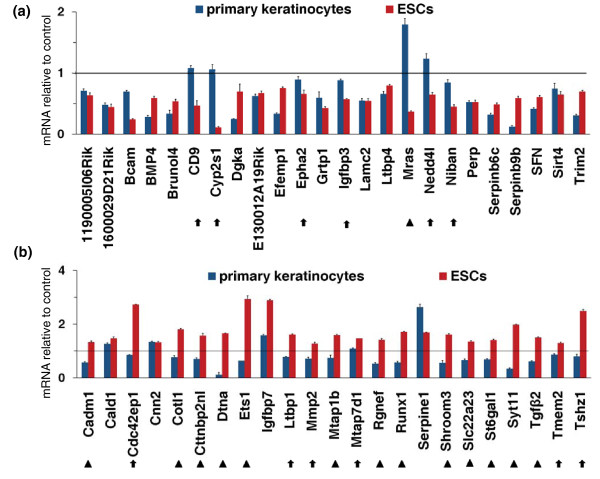
**ESC-specific targets of Klf5**. qPCR analysis of expression changes of Klf5 target genes upon Klf5 KD in primary keratinocytes compared to results obtained in ESCs. Klf5 or NS siRNA were transfected in both primary keratinocytes, and ESCs and gene expression levels were measured 12 hr after transfection. Probe sets of both genes induced **(a) **and repressed **(b) **by Klf5 in ESCs were analyzed. Arrows indicate genes regulated only in ESCs; arrowheads indicate genes showing an opposite change upon Klf5 KD in ESCs and primary keratinocytes. All data are expressed as fold changes relative to siNS transfected cells. Error bars represent SD of triplicates.

Next, we asked the question of the specificity of Klf5. Previous works demonstrated that Klf5 is highly expressed in skin [19] and that alterations in Klf5 expression level may affect the epidermis-differentiated phenotype [29], suggesting that Klf5 could regulate the regenerative potential of stem cells in the epidermis. We analyzed expression changes of the 53 qPCR-validated Klf5 targets upon Klf5 KD in primary keratinocytes (Additional file 10). In fact, we found that the transcripts of six genes expressed in ESCs were not detectable in primary keratinocytes, both in Klf5 KD and control cells, whereas there were 21 of 47 genes similarly regulated by Klf5 in primary keratinocytes and ESCs. On the other hand, 14 of 47 genes showed opposite changes in ESCs versus keratinocytes, while 12 of 47 genes were regulated by Klf5 only in ESCs (Figure 4). Although the extent of Klf5 suppression is different in ESCs versus keratinocytes, the comparison of expression profiles of these two cells indicated a cell type-specific gene regulation by Klf5.

**Figure 5:**
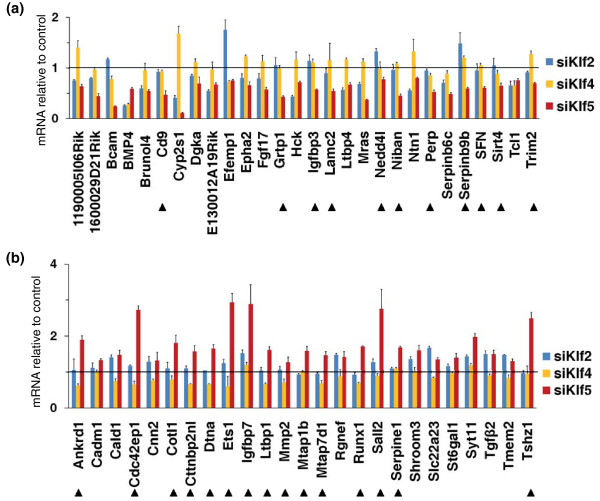
**Klf5-specific targets**. Comparison of gene expression changes following KD of Klf2, Klf4 or Klf5 in ESCs. Klf2, Klf4, Klf5 or NS siRNA were transfected in ESCs. RNA samples were collected 12 hr after transfection and subjected to qPCR. **(a) **Klf5-activated genes. **(b) **Klf5-repressed genes. Arrowheads indicate the expression changes induced by Klf5 only. All data are expressed as fold changes relative to siNS transfected cells. Error bars represent SD of triplicates.

A further aspect of the specificity of the Klf5-based regulation concerns the possibility that Klf2, Klf4 and Klf5 have redundant functions by binding and thus regulating common targets in ESCs [15]. To address this point, we investigated gene expression changes of the 53 qPCR-validated Klf5 targets, following KD of each single Klf. We transfected Klf2 or Klf4 siRNA in ESCs, and 12 hr after transfection we found that the level of Klf2 and Klf4 were significantly reduced, although with different extent of KD (Additional file 10). In these conditions, we found that only a low percentage (<10%) of examined genes showed the same trend upon KD of all these Klfs (Figure 5). The same low percentage was observed by comparing Klf5- versus Klf4-regulated genes. Instead, we found that the genes that showed the same behavior upon Klf2 and Klf5 KD represent about 40%. Finally, we observed that about 45% of the genes show a Klf5-specific gene regulation, different from that dependent on Klf2 or Klf4 KD (Figure 5), indicating that some redundancy could exist in the genes controlled by Klf5 and Klf2, rather than Klf4.

## Discussion

In the past 3 years, several reports have shown Klf5 to be an important player regulating ESC self-renewal, pluripotency and embryonic development with a unique requirement of Klf5 in inner cell mass (ICM) establishment compared to the other ESC-specific Klfs [16,17,30-32]. Although the pivotal role of Klf5 in ESC self-renewal and ICM development has been at least in part elucidated, its mechanism of action and the genes that Klf5 controls are still not completely understood. In the present study, we analyzed Klf5 regulatory targets in ESCs by comparing microarray and ChIP-seq analysis. We identified 74 Klf5 target genes whose expression is activated by Klf5 and 239 whose expression is repressed. Among them, we found 161 genes bearing Klf5 binding sites within 100 kb from the gene boundaries and that can be considered putative primary targets of Klf5.

We have shown that Klf5 controls genes that play a crucial role in ESCs such as Tcl1 [8,22], BMP4 [23] and Nr0b1 [24], and thus it may be required in ESCs to maintain pluripotency by activating expression of these self-renewal promoting genes and by simultaneously inhibiting expression of differentiation promoting genes such as TGFβ2 [33], Otx2, Pitx2 [8] and GDNF [34]. To further support the role of Klf5 in the control of regulators of the ESC phenotype, we have explored the effects of the suppression of 23 genes target of Klf5. Among these 23 genes, we identified 8 genes whose KD induces the loss of ESC undifferentiated state even in the presence of LIF. The eight identified genes encode cell adhesion molecules (Bcam, Perp), two protein kinases (Hck, Dgka), a growth factor binding protein (Igfbp3), an endoplasmic reticulum protein (Niban) and two proteins with still unknown function (1600029D21Rik, E130012A19Rik). Such a variety of molecules indicates that the ability of Klf5 to maintain the ESC undifferentiated state results from the concomitant regulation of a wide range of cellular activities. These eight genes showed the same trend of Klf5 expression during ESC differentiation and, moreover, a different transcriptional control by Klf2, Klf4 and Klf5 with Igfb3, Niban and Perp responding only to Klf5. Among these proteins, Hck has been shown to be involved in gp130-mediated signaling of LIF, since introduction of constitutively activated Hck alleviates the requirement of ESCs for LIF to remain undifferentiated [35]. Our data suggest that Klf5 is required for proper control of the expression of ESC-specific genes as well as genes whose transcription seems to be induced upon ESC differentiation. Among the genes suppressed by Klf5 in undifferentiated ESCs, we found Serpine1, also known as plasminogen activator inhibitor-1 (PAI-1). Serpine1 is a downstream target of TGFβ1 and is induced upon ESC differentiation by TGFβ1 stimulation [36]. We have found that ectopic expression of Serpine1 resulted in an impairment of the ESC phenotype by promoting the appearance of differentiation markers even in the presence of LIF. This phenotype mimics the effect of Klf5 KD as well as of Klf5 KO ESCs [16,17]. Although a redundant function was suggested for Klf2, Klf4 and Klf5 [15], we have shown that Serpine1 is regulated specifically by Klf5 and not by Klf2 and Klf4 suppression. This specific control by Klf5 could explain the inability of Klf2 and Klf4 to compensate for the loss of Klf5 function in ICM establishment.

Klf5 has been described to play a role in various biological processes, such as the control of the stress response in the myocardium [37] and the induction of somatic cell reprogramming [14]. Furthermore, Klf5 has many roles in regulating cell cycle, development, proliferation, apoptosis and tumorigenesis [18]. Interestingly, Sur et al. [29] described a role of Klf5 in the regulation of the keratinocyte differentiation program and thus in the regenerative potential of stem cells in the epidermis. By investigating changes in expression profile of Klf5 target genes both in ESCs and primary keratinocytes, we have found that Klf5 may work as a repressor or activator in a context-specific manner. In fact, about half of the examined genes seem to be regulated by Klf5 in an ESC-specific manner, and in many cases the effect of Klf5 KD in ESCs and primary keratinocytes was opposite. This different regulation by Klf5 could be due to a different accessibility to transcriptional regulators as a consequence of chromatin folding and/or to the interaction with cell-specific transcriptional complexes.

Recently, Jiang et al. [15] proposed that Klf5 has a redundant function with Klf2 and Klf4 in ESCs. The same authors showed that there is a greater overlap between genomic binding sites of Klf2 and Klf4 than with Klf5. Accordingly, we found that about 45% of the examined genes are significantly regulated by Klf2 and Klf5. Interestingly, we also observed that almost half of the examined genes appear to be regulated specifically by Klf5. This can explain the unique requirement of Klf5 for derivation of ESCs and ICM proper development [17], in contrast to the dispensable role of Klf2 and Klf4 in these processes [31,32].

## Conclusions

Numerous results indicate that Klf5 plays an important role in maintaining ESC pluripotency and in governing ESC fate decisions. In this work, we have explored the mechanisms through which this transcription factor regulates ESC functions. We have identified a likely complete set of genes, putative primary targets of Klf5 in ESCs, by comparing the early changes of the gene expression profile induced by Klf5 KD and the results of ChIP-seq analysis for Klf5 binding sites. About half of these genes seem to be regulated by Klf5 in an ESC-specific manner, and Klf5-dependent gene regulation in ESCs appears to be different from that based on Klf2 and Klf4, two other members of the Krüppel-like family, previously involved in ESC functions. Finally, we demonstrated that suppression or constitutive expression of Klf5 target genes clearly impair the ESC undifferentiated state. These results contribute to the understanding of the regulatory role of Klf5 in ESCs and suggest a high hierarchical role in these cells for this transcription factor.

## Materials and methods

### Plasmid construction

Serpine1 and Runx1 cDNA were derived from pSport vector (NIH Mammalian Gene Collection, Open Biosystems, National Institutes of Health, Bethesda, MD, USA) by PCR [16] with the following oligonucleotides:

*Hin*dIII-Serpine1: 5'-GATGACAAGCTTCAGATGTCTTCAGCCCTTGCTTGCCTCATCC-3'

*Not*I-Serpine1: 5'-GGCGATGAGCGGCCGCTCAAGGCTCCATCACTTGGCCCATGAAGAGG-3'

*Hin*dIII-Runx1: 5'-GATGACAAGCTTGCTTCAGACAGCATTTTTGAGTCATTTCCTTCATATCC-3'

*Not*I-Runx1: 5'-GGCGATGAGCGGCCGCTCAGTAGGGCCGCCACACGGCCTCCTCC-3'

Next, these cDNAs were cloned downstream FLAG-tag in the p-CBA-FLAG vector by using *Hin*dIII and *Not*I restriction sites.

### Cell culture, transfection, differentiation and alkaline phosphatase staining

E14Tg2a (BayGenomics, San Francisco, CA, USA) mouse ESCs were maintained on feeder-free, gelatin-coated plates in the following medium: GMEM (Glasgow Minimum Essential Medium) (Sigma, St. Louis, MO, USA) supplemented with 2 mM glutamine, 100 U/ml penicillin/streptomycin, 1 mM sodium pyruvate, 1× nonessential amino acids (all from Invitrogen, Carlsbad, CA, USA), 0.1 mM β-mercaptoethanol (Sigma), 10% fetal bovine serum (HyClone Laboratories, Logan, UT, USA), and 10^3 ^U/ml leukemia inhibitory factor (LIF; Millipore, Billerica, MA, USA). Neural differentiation was induced as previously described [16]. Briefly, undifferentiated ESCs were trypsinized into a single-cell suspension and plated at low density (1-5 × 10^3 ^cells/cm^2^) on gelatin-coated dishes in the following medium: knockout Dulbecco's minimal essential medium supplemented with 10% knockout serum replacement (both from Invitrogen), 0.1 mM β-mercaptoethanol (Sigma), 2 mM glutamine (Invitrogen), 100 U/mL penicillin/streptomycin (Invitrogen). Medium was changed on alternate days.

Primary mouse keratinocytes were isolated from 2-day-old Swiss ICR(CD-1) mice (Harlan Laboratories, Correzzana, Italy) and cultured as previously described [38]. Transfections were performed 5 days after plating.

Transfection of expression plasmids and shRNA plasmids (Open Biosystems; see Additional file 11) both in ESCs and in primary keratinocytes were performed using Lipofectamine 2000 (Invitrogen) following the manufacturer's instructions. To generate the stable cell lines, E14Tg2a cells were transfected with shRNA plasmids and recombinant clones were selected with Puromycin (Sigma).

For alkaline phosphatase staining, ESCs were cultured at clonal density (30 cells/cm^2^). The cells were fixed in 10% cold neutral formalin buffer (10% formalin, 110 mM Na_2_HPO_4_, 30 mM NaH_2_PO_4 _H_2_O) for 15 min and then rinsed in distilled water for 15 min. The staining was obtained by incubation for 45 min at room temperature with the following staining solution: 0.1 M Tris·HCl, 0.01% naphthol AS MX-PO_4 _(Sigma), 0.4% *N, N*-dimethylformamide (Sigma), 0.06% red violet LB salt (Sigma).

### RNA isolation and qPCR

RNA from ESCs and primary keratinocytes was isolated by using the Tri reagent (Sigma) and then reverse transcribed using MuMLV-RT (New England Biolabs, Ipswich, MA, USA) according to the manufacturer's instructions. Real-time RT-PCR was performed using PRISM 7900HT Sequence Detection System (Applied Biosystems, Foster City, CA USA) and Power SYBR Green PCR Master Mix (Applied Biosystems). Gene-specific primers used for amplification are listed in Additional file 12.

### Microarrays and bioinformatics analysis

Total RNA from ESCs was isolated with Tri reagent (Sigma) and further purified with the Qiagen column kit (Qiagen, Milan, Italy). Then samples from three independent experiments were sent to Coriell Genotyping and Microarray Center (Coriell Institute for Medical Research, Camden, NJ, USA), where, after proper sample processing, cRNA were hybridized with the Affymetrix Mouse Genome 430 2.0 Array (Affymetrix, Santa Clara, CA, USA).

For bioinformatics analysis, raw probe intensities for each of the hybridized microarrays were normalized to gene expression levels using the dChip algorithm [39]. To identify genes significantly responding in the experiment, we computed the *P *values and false discovery rate (FDR). A total of 313 probes, corresponding to 313 different transcripts, have been identified (FDR < 0.1 corresponding to *P *< 0.005) that responded significantly.

### Preparation of cell lysates and Western blot analysis

ESCs and primary keratinocytes were lysed in a buffer containing 1 mM EDTA, 50 mM Tris·HCl, pH 7.5, 70 mM NaCl, 1% Triton protease inhibitor cocktail (Sigma) and analyzed by Western blot analysis. The following primary antibodies were used: rabbit anti-Klf5 (Santa Cruz Biotechnology, Santa Cruz, CA, USA), rabbit anti-Nanog (Calbiochem, San Diego, CA, USA), mouse anti-Oct3/4 (Santa Cruz Biotechnology), mouse anti-GAPDH (Santa Cruz Biotechnology) and mouse anti-FLAG (Sigma). Antibody-protein complexes were detected by HRP-conjugated antibodies and ECL (both from Amersham Pharmacia, Milan, Italy).

### Chromatin immunoprecipitation

For ChIP-seq analysis, ESCs stably transfected with FLAG-Klf5 were cross-linked with 1% formaldehyde for 10 min at room temperature, and formaldehyde was then inactivated by the addition of 125 mM glycine. Then the chromatin was sonicated to an average DNA fragment length of 200 to 500 bp. Soluble chromatin extracts were immunoprecipitated using the mouse monoclonal anti-FLAG (Sigma) or mouse IgG (Santa Cruz Biotechnology) as control. Then samples from two independent experiments were sent to the DNA sequencing service of EMBL (Heidelberg, Germany) and subjected to high-throughput sequencing with Illumina Genome Analyzer platform (Illumina, San Diego, CA, USA).

For ChIP-qPCR, samples were prepared as described above. Supernatant obtained without antibody was used as an input control. qPCR analyses were performed using the ABI PRISM 7900HT sequence detection system and SYBR Green PCR Master Mix (Applied Biosystems). Primers used for ChIP-qPCR are listed in Additional file 13.

The amount of precipitated DNA was calculated relative to the total input chromatin and expressed as the fold enrichment relative to total input according to the following formula [40]: fold enrichment = 2{Delta}Ct × 10, where {Delta}Ct = Ct(input) - Ct(immunoprecipitation), where Ct refers to cycle threshold.

### ChIP-seq bioinformatics analysis

More than 13 million sequences were produced and aligned to the mouse genome (version m37) masked for DNA repeat by using Bowtie tool version 0.9.9.2 (Center for Bioinformatics and Computational Biology, Institute for Advanced Computer Studies, University of Maryland, College Park, MD, USA) [41]. About 50% of sequences were univocally aligned, and the resulting coordinates were fed to MACS software version 1.3.5(Department of Biostatistics and Computational Biology, Dana-Farber Cancer Institute and Harvard School of Public Health, Boston, MA USA) [42] to detect genomic regions enriched for multiple overlapping DNA fragments (peaks) that we considered as putative binding sites. False discovery rate (FDR) was estimated by MACS, by comparing the peaks from anti-FLAG samples with those from control (anti-IgG ChIP) at the same *P *value cutoff. Motif analysis was performed by using CisFinder tool (Developmental Genomics and Aging Section, Laboratory of Genetics, National Institute on Aging, NIH, Baltimore, MD, USA) [43] on 200-bp sequences centered at the expected binding site indicated by peak summit calculated using the MACS tool. Flanking sequences 1000 bp away from the peak summit have been used as control sequences.

## Abbreviations

(AP): Alkaline phosphatase; (BCAM): basal cell adhesion molecule; (BMP4): bone morphogenetic protein 4; (KLF): Krüppel-like factor; (CHIP): chromatin immunoprecipitation; (DGKα): diacylglycerol kinase α; (ESC): embryonic stem cell; (GDNF): glial cell-derived neurotrophic factor; (HCK): hemopoietic cell kinase; (ICM): inner cell mass; (IGFBP3): insulin-like growth factor binding protein 3; (KD): knockdown; (KO): knockout; (LIF): leukemia inhibitory factor; (NR0B1): nuclear receptor subfamily 0, group B, member 1; (OCT3/4): octamer-binding protein 3; (OTX2): orthodenticle homeobox 2; (PITX2): paired-like homeodomain 2; (RUNX1): runt-related transcription factor 1; (SHRNA): short hairpin RNA; (SIRNA): short interfering RNA; (Sox2): SRY-box containing gene 2; (TCL1): T-cell lymphoma breakpoint 1; (TGFβ2): transforming growth factor β2.

## Authors' contributions

SP was responsible for the study's conception and design, collection and assembly of data, data analysis and interpretation, and manuscript writing; LC was responsible for collection and assembly of data; CT was responsible for collection and assembly of data; FP was responsible for collection and assembly of data; SC was responsible for collection and assembly of data; LA was responsible for collection and assembly of data; DA was responsible for collection and assembly of data; VDS was responsible for data analysis and interpretation; LP was responsible for manuscript writing; TR was responsible for conception and design, data analysis and interpretation, and manuscript writing. All authors read and approved the final manuscript.

## Supplementary Material

Additional file 1 **Additional Table 1**. Gene expression profile by microarray analysis upon Klf5 KD in ESCs. Fold change is calculated by comparing data from siKlf5 cells over the control (siNS). Cut-off >1.25- and <0.75-fold changes were used. Probes with FDR <0.1 were selected. Click here for file

Additional file 2 **Additional Figure 1**. GO analysis of Klf5 targets and Klf expression profile during ESC differentiation. **(a) **Gene ontology (GO) annotation of the selected probe sets according to DAVID "Biological Process Classification" tool (Laboratory of Immunopathogenesis and Bioinformatics, Clinical Services Program, SAIC-Frederick, Inc., National Cancer Institute at Frederick, Frederick, MD, USA). **(b) **Expression levels of Klf2, Klf4 and Klf5 were measured by qPCR in undifferentiated (t0) and 4-day (4d) and 7-day (7d) differentiated ESCs. The data are represented as fold changes relative to undifferentiated cells. Click here for file

Additional file 3 **Additional Table 2**. Microarray data were compared with published results. Column siKlf5/siNS is referred to our microarray data (see also Additional Table 1). Differences with published data are highlighted in red. dw: downregulated gene, up: upregulated gene; na: data not available. Click here for file

Additional file 4 **Additional Figure 2**. ChIP-seq validation by ChIP-qPCR. **(a) **Expression level of FLAG-Klf5 stable clone pools used to prepare chromatin for ChIP-seq experiment. Western blot was stained with an anti-FLAG and anti-Klf5. **(b) **List of peaks validated by ChIP-qPCR. Peaks with different numbers of tags were chosen. Peak location are indicated (chr, chromosome). **(c) **ChIP-seq validation was performed by ChIP-qPCR using anti-FLAG antibody and IgG, as control, with extracts derived from FLAG-Klf5 and Mock transfected ESCs. The data are expressed as the amount of precipitated DNA calculated relative to the total input chromatin. Samples from 1 to 15 correspond to regions close to the following genes: Agap1, Lamc2, Fcgr3, 170009P17Rik, Tgfβ2, Smx16, Nlgn1, Epha2 (upstream region), Epha2 (downstream region), Igfbp7, Serpine1, Cyp2s1, 4930467E23Rik, AC152164, Inpp4b, respectively. Three different control regions were chosen (samples 16, 17 and 18): chr1:10573933-10573984, chr1:71481391-71481461, chr3:12034661-12034625, respectively, where no significant peaks were found. Bars represent SD of triplicates. Click here for file

Additional file 5 **Additional Table 3**. Results of ChIP-seq analysis. The putative binding sites of Klf5 are reported with relative number of tags for each peak and FDR. Match with gene microarray data is shown. The distance from the 5' and 3' boundaries of the Klf5-regulated genes is indicated. Click here for file

Additional file 6 **Additional Figure 3**. Klf5 binding motifs identified with CisFinder via 200-bp sequences centered at binding peaks (E-score > 22). Click here for file

Additional file 7 **Additional Figure 4**. qPCR validation of microarray data. Sixty Klf5 target genes were analyzed by qPCR to confirm the microarray data. Probe set of both downregulated **(a) **and upregulated **(b) **genes upon Klf5 KD is shown. Black bars represent not validated probes. The data are expressed as fold change relative to siNS transfected cells. Validated probes showed a *P *< 0.01. Click here for file

Additional file 8 **Additional Figure 5**. KD of a subset of Klf5-target genes. **(a) **ESCs were stably transfected with shRNA plasmids for selected Klf5-target genes or with control shRNA (shGFP) and KD was verified by qPCR. The results are represented as fold changes relative to shGFP-transfected cells. SD of triplicates is reported. **(b) **Percentage of undifferentiated (blue) and differentiated (red) colonies observed by AP staining upon KD of eight Klf5-target genes with a second independent shRNA. *P < 0.01. **(c) **Expression levels of Oct3/4 and Nanog upon KD of eight Klf5-target genes with a second independent shRNA. The data are represented as fold changes relative to shGFP-transfected cells. Click here for file

Additional file 9 **Additional Figure 6**. Expression of early differentiation markers of endoderm (Sox17), mesoderm (Brachyury) and ectoderm (Fgf5) upon KD of eight Klf5-target genes. Click here for file

Additional file 10 **Additional Figure 7**. Klf5 KD in primary keratinocytes and Klf2 and Klf4 KD in ESCs. **(a) **Klf5 or NS siRNA were transfected in primary keratinocytes and Klf5 expression level was measured 12 hr after transfection by Western blot with anti-Klf5 antibody. **(b) **Expression levels of Klf2, Klf4 and Klf5 were measured by qPCR in ESCs 12 hours after siRNA transfection. The results are represented as fold changes. Bars represent SD of triplicates. *P *< 0.01. Click here for file

Additional file 11 **Additional Table 4**. Sequences of shRNAs. Click here for file

Additional file 12 **Additional Table 5**. Primers used for qPCR. Click here for file

Additional file 13 **Additional Table 6**. Primers used for ChIP-qPCR. Click here for file
