# Supplementary figures and images for: Direct targets of Klf5 transcription factor contribute to the maintenance of mouse embryonic stem cell undifferentiated state
Source: BMC Biol. 2010 Sep 27;8:128. doi: 10.1186/1741-7007-8-128 (PMC2955566; doi:10.1186/1741-7007-8-128)

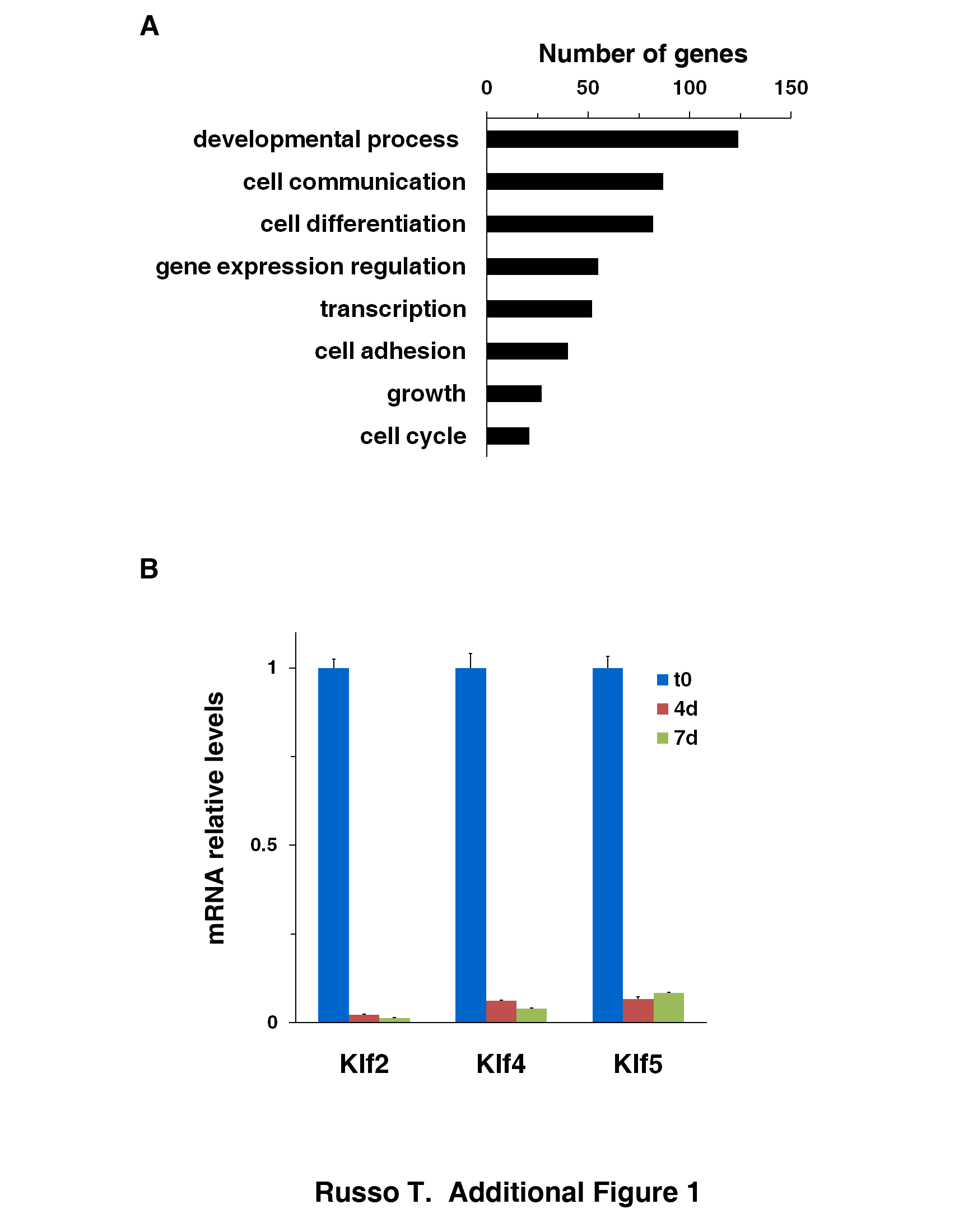

Supplement: Additional file 2 — Additional Figure 1. GO analysis of Klf5 targets and Klf expression profile during ESC differentiation. (a) Gene ontology (GO) annotation of the selected probe sets according to DAVID "Biological Process Classification" tool (Laboratory of Immunopathogenesis and Bioinformatics, Clinical Services Program, SAIC-Frederick, Inc., National Cancer Institute at Frederick, Frederick, MD, USA). (b) Expression levels of Klf2, Klf4 and Klf5 were measured by qPCR in undifferentiated (t0) and 4-day (4d) and 7-day (7d) differentiated ESCs. The data are represented as fold changes relative to undifferentiated cells. [file 1741-7007-8-128-S2.TIFF]

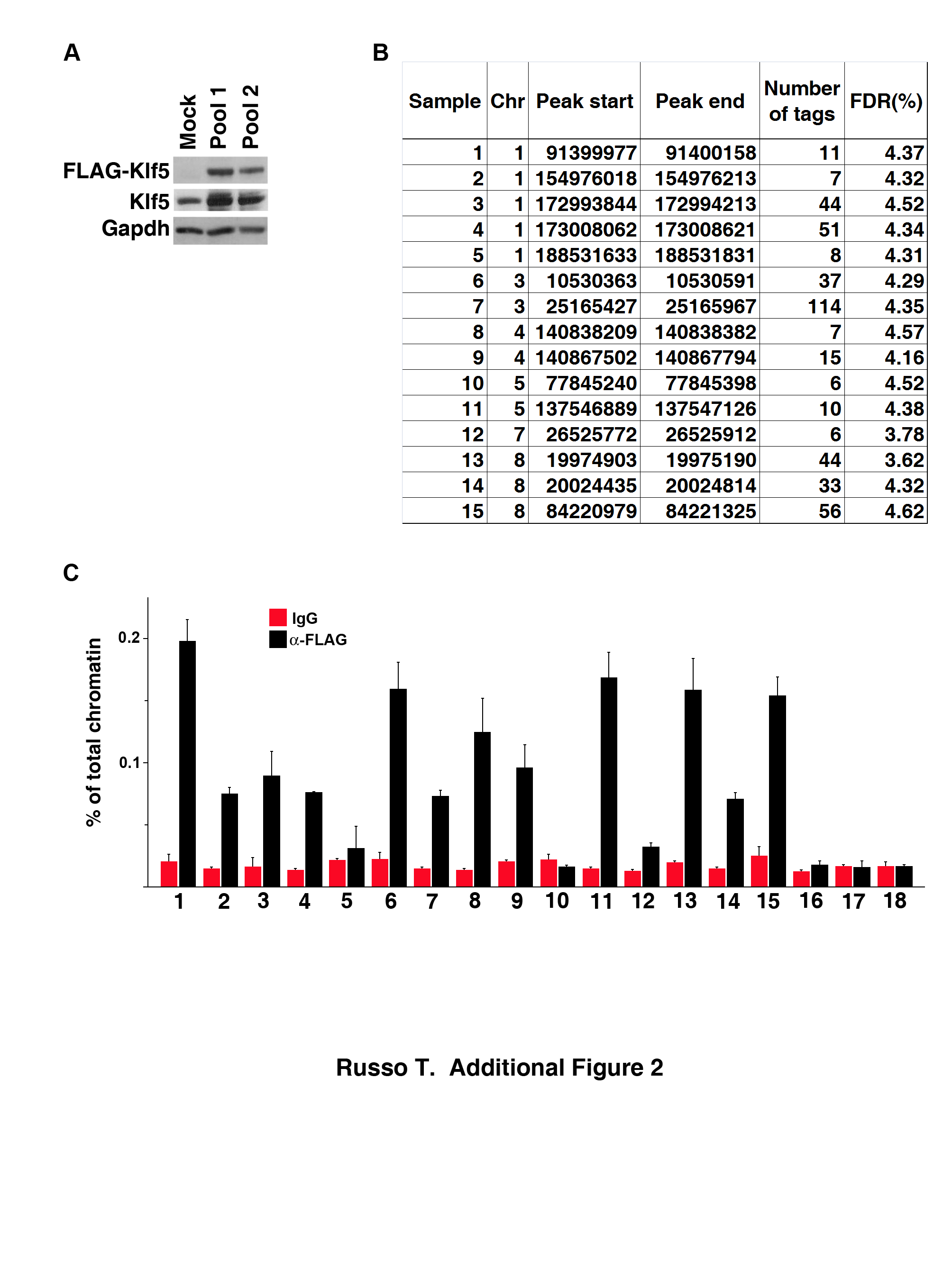

Supplement: Additional file 4 — Additional Figure 2. ChIP-seq validation by ChIP-qPCR. (a) Expression level of FLAG-Klf5 stable clone pools used to prepare chromatin for ChIP-seq experiment. Western blot was stained with an anti-FLAG and anti-Klf5. (b) List of peaks validated by ChIP-qPCR. Peaks with different numbers of tags were chosen. Peak location are indicated (chr, chromosome). (c) ChIP-seq validation was performed by ChIP-qPCR using anti-FLAG antibody and IgG, as control, with extracts derived from FLAG-Klf5 and Mock transfected ESCs. The data are expressed as the amount of precipitated DNA calculated relative to the total input chromatin. Samples from 1 to 15 correspond to regions close to the following genes: Agap1, Lamc2, Fcgr3, 170009P17Rik, Tgfβ2, Smx16, Nlgn1, Epha2 (upstream region), Epha2 (downstream region), Igfbp7, Serpine1, Cyp2s1, 4930467E23Rik, AC152164, Inpp4b, respectively. Three different control regions were chosen (samples 16, 17 and 18): chr1:10573933-10573984, chr1:71481391-71481461, chr3:12034661-12034625, respectively, where no significant peaks were found. Bars represent SD of triplicates. [file 1741-7007-8-128-S4.TIFF]

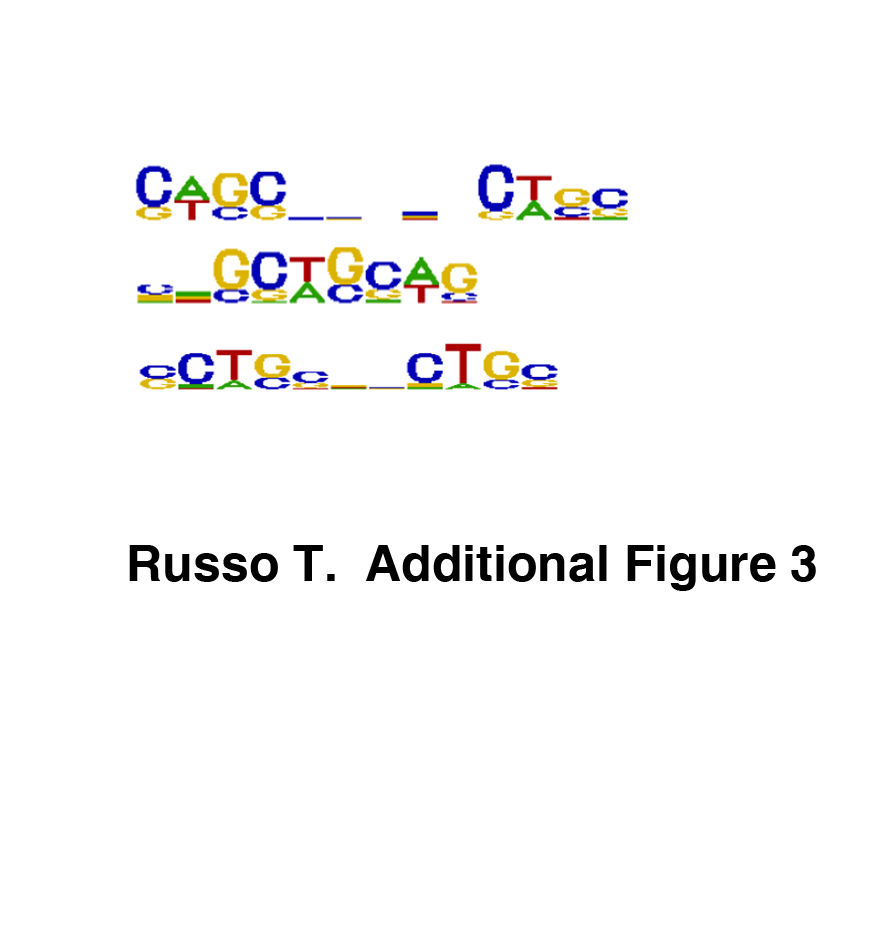

Supplement: Additional file 6 — Additional Figure 3. Klf5 binding motifs identified with CisFinder via 200-bp sequences centered at binding peaks (E-score > 22). [file 1741-7007-8-128-S6.TIFF]

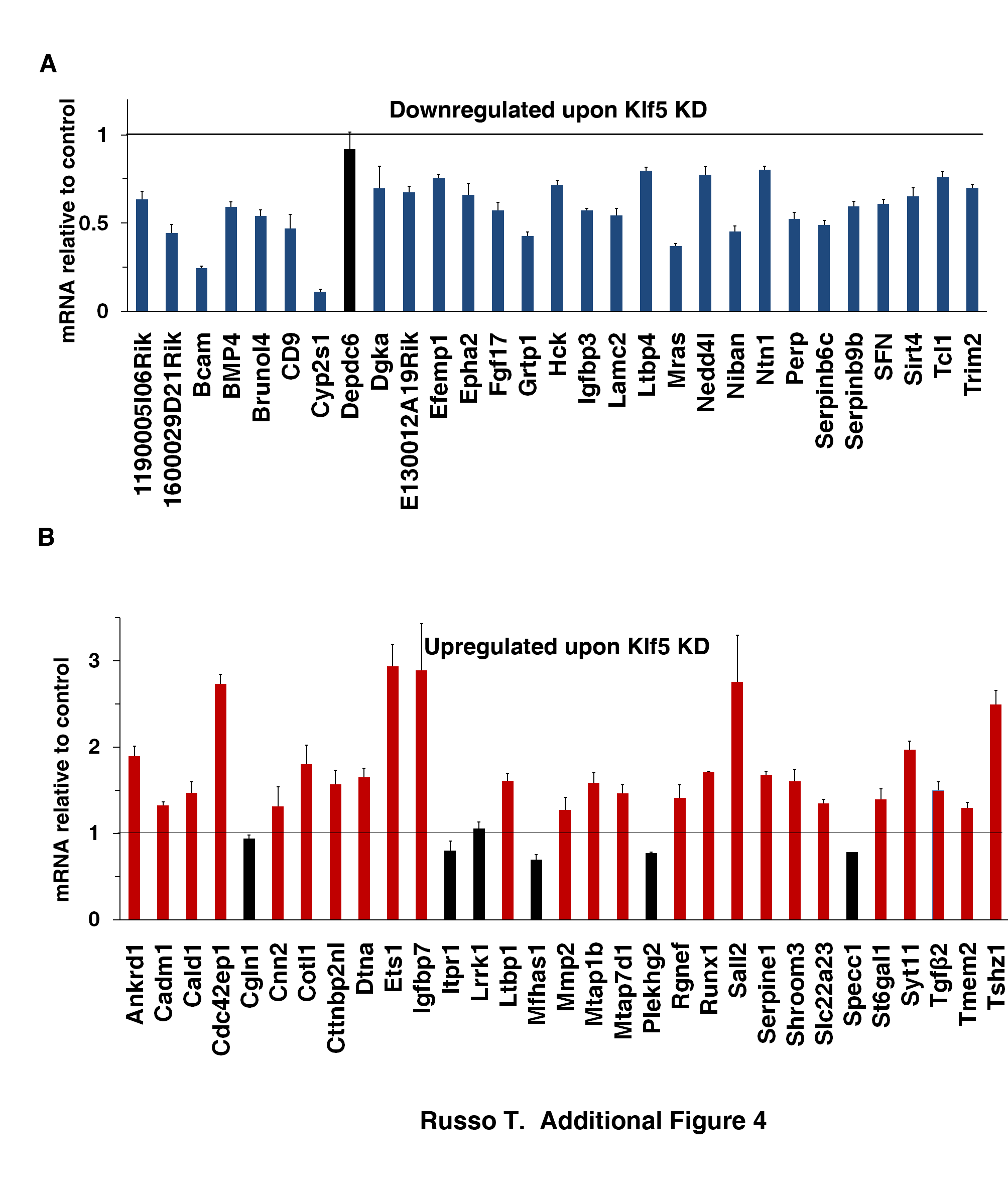

Supplement: Additional file 7 — Additional Figure 4. qPCR validation of microarray data. Sixty Klf5 target genes were analyzed by qPCR to confirm the microarray data. Probe set of both downregulated (a) and upregulated (b) genes upon Klf5 KD is shown. Black bars represent not validated probes. The data are expressed as fold change relative to siNS transfected cells. Validated probes showed a P < 0.01. [file 1741-7007-8-128-S7.TIFF]

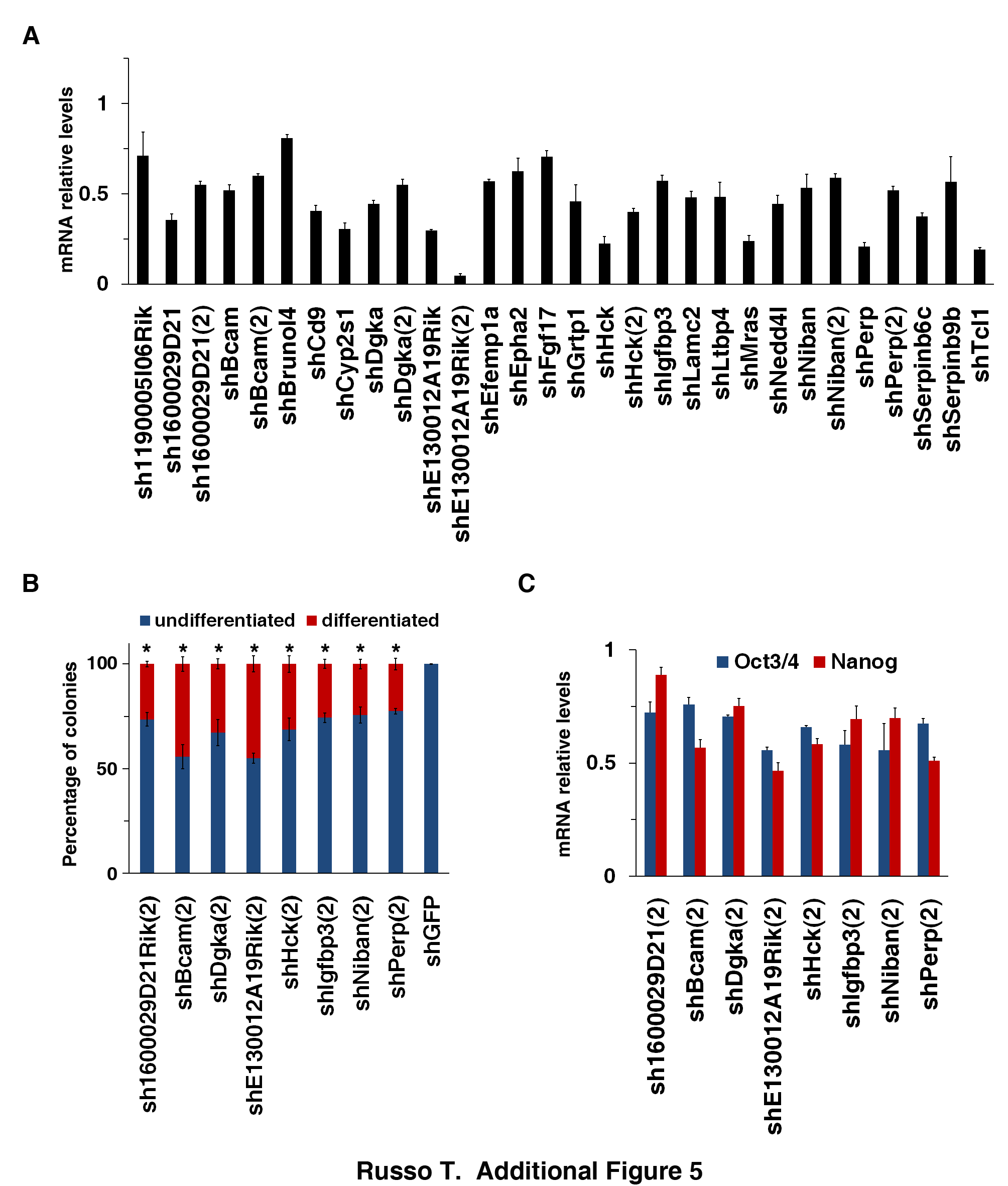

Supplement: Additional file 8 — Additional Figure 5. KD of a subset of Klf5-target genes. (a) ESCs were stably transfected with shRNA plasmids for selected Klf5-target genes or with control shRNA (shGFP) and KD was verified by qPCR. The results are represented as fold changes relative to shGFP-transfected cells. SD of triplicates is reported. (b) Percentage of undifferentiated (blue) and differentiated (red) colonies observed by AP staining upon KD of eight Klf5-target genes with a second independent shRNA. *P < 0.01. (c) Expression levels of Oct3/4 and Nanog upon KD of eight Klf5-target genes with a second independent shRNA. The data are represented as fold changes relative to shGFP-transfected cells. [file 1741-7007-8-128-S8.TIFF]

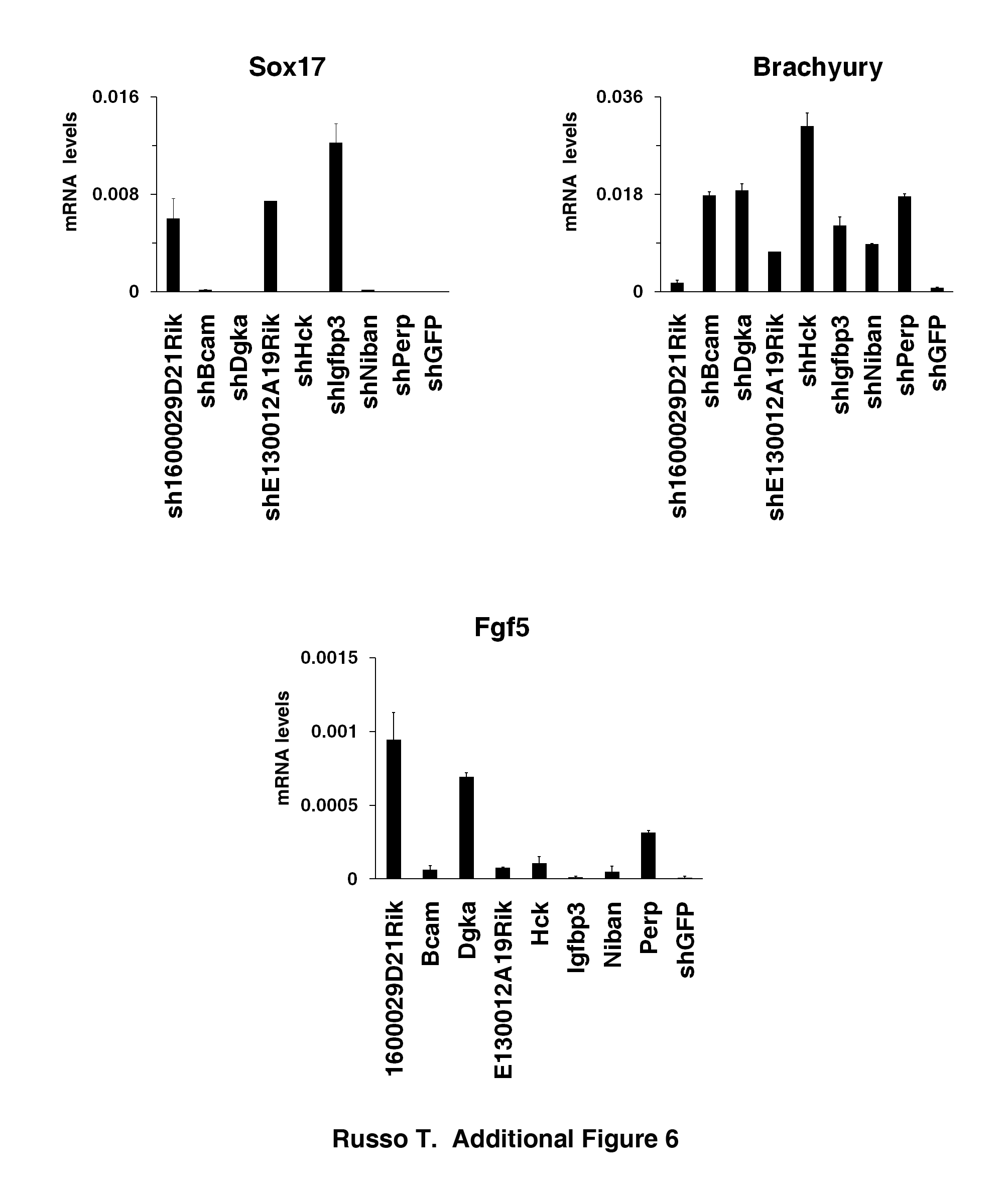

Supplement: Additional file 9 — Additional Figure 6. Expression of early differentiation markers of endoderm (Sox17), mesoderm (Brachyury) and ectoderm (Fgf5) upon KD of eight Klf5-target genes. [file 1741-7007-8-128-S9.TIFF]

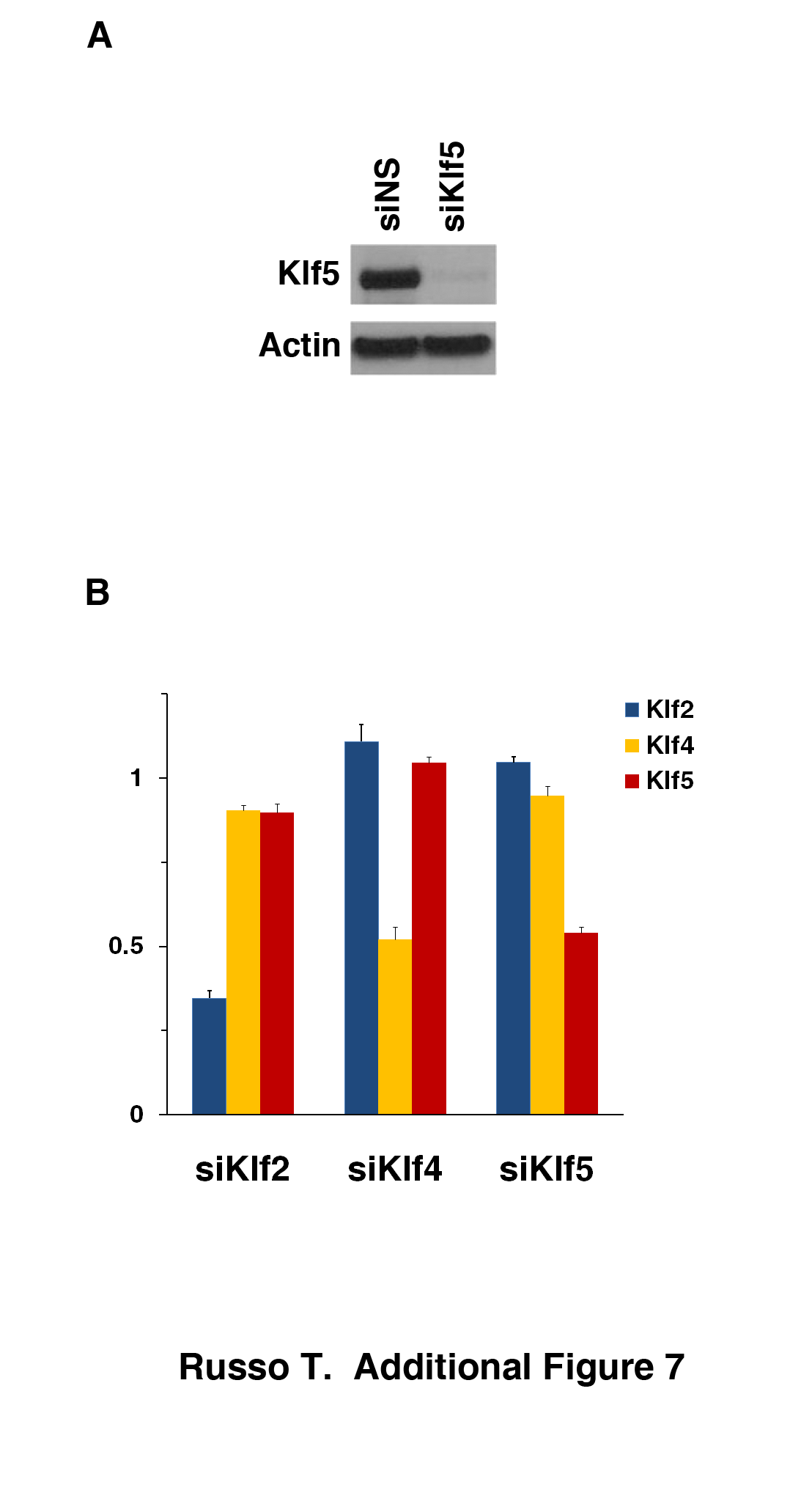

Supplement: Additional file 10 — Additional Figure 7. Klf5 KD in primary keratinocytes and Klf2 and Klf4 KD in ESCs. (a) Klf5 or NS siRNA were transfected in primary keratinocytes and Klf5 expression level was measured 12 hr after transfection by Western blot with anti-Klf5 antibody. (b) Expression levels of Klf2, Klf4 and Klf5 were measured by qPCR in ESCs 12 hours after siRNA transfection. The results are represented as fold changes. Bars represent SD of triplicates. P < 0.01. [file 1741-7007-8-128-S10.TIFF]
