# Supplementary material for: Direct targets of Klf5 transcription factor contribute to the maintenance of mouse embryonic stem cell undifferentiated state
Source: BMC Biol. 2010 Sep 27;8:128. doi: 10.1186/1741-7007-8-128 (PMC2955566; doi:10.1186/1741-7007-8-128)
Supplement: Additional file 11 — Additional Table 4. Sequences of shRNAs. [file 1741-7007-8-128-S11.DOC]

| **Gene name** | **shRNA sequence*** |
| --- | --- |
| Klf5 | CCGTATCCACTTCTGCGATTAT |
| 1190005I06Rik | CCCTGAGCGAGATTTATAAATA |
| 1600029D21Rik | AGGAGGGAATGATCCTTCTTTA |
| 1600029D21Rik | CCGACAGCAGTGTCTACTATAA |
| Bcam | CCCTCAGCCCATCATTTGTGAA |
| Bcam | CTGGCTGCAGAGTGGAGGATTA |
| Brunol4 | ACGCGACTACATGCACATATAT |
| CD9 | ACCAGACTCTAGACATAAGATA |
| Cyp2s1 | CCGATGTTCTGTCTACTCCCAT |
| Dgka | AGGTACCTAGGATTTGAACAAT |
| Dgka | CGCCAGGGCTCAGATTCTTTAA |
| E130012A19Rik | ACCGCTCGAAACTTTGGTGTTA |
| E130012A19Rik | ATGGGTCAACTTGGAAGAACTA |
| Efemp1 | CGCATAATGTGTGCCAAGATAT |
| Epha2 | AGCCAGTTTAGCCACCACAATA |
| Fgf17 | AGGAGAATCACCCGTCTCCTAA |
| Grtp1 | CCCACAACGTTCCTGCTGTAAA |
| Hck | GGAAACAATCAACCAGTCA |
| Hck | AGGTTAGCAAAGAAATGCTGAA |
| Igfbp3 | CGCCAAGATGGATGTCATCAAA |
| Igfbp3 | CGCCGCCCTTCCAAAGGCAGAA |
| Lamc2 | ACCAGCTGAGTTATTTCGAATA |
| Ltbp4 | CCGCTGCCCATTCTTCGAAATA |
| Mras | CCCAAATACAATATCCCATATA |
| Nedd4l | CGCTCGCCAACAGTAACTTTAT |
| Niban | CCCTCTCACTGTAGAGAAT |
| Niban | AGCCCTCTCACTGTAGAGAATA |
| Perp | ACCTGCTGTTAATTACATCTAT |
| Perp | CCCAGATCATCTCCCTGGTAAT |
| Serpinb6c | CGGAAATAACTCATGAGAAATT |
| Serpinb9b | CGCTATGCCACTGATACCCATA |
| Tcl1 | CCGGCTGGATTTAATAAAGCTT |

**Additional Table 5:** Sequences of shRNAs.

***** All from Open Biosystems
