# Supplementary material for: Direct targets of Klf5 transcription factor contribute to the maintenance of mouse embryonic stem cell undifferentiated state
Source: BMC Biol. 2010 Sep 27;8:128. doi: 10.1186/1741-7007-8-128 (PMC2955566; doi:10.1186/1741-7007-8-128)
Supplement: Additional file 12 — Additional Table 5. Primers used for qPCR. [file 1741-7007-8-128-S12.DOC]

**Additional Table 5:** Primers used for qPCR

| **Gene** | **Forward primer** | **Reverse primer** |
| --- | --- | --- |
| Klf5 | GGTCCAGACAAGATGTGAAATGG | TTTATGCTCTGAAATTATCGGAACTG |
| Nanog | TCAGAAGGGCTCAGCACCA | GCGTTCACCAGATAGCCCTG |
| Oct3/4 | AACCTTCAGGAGATATGCAAATCG | TTCTCAATGCTAGTTCGCTTTCTCT |
| Fgf5 | CCTCATCTTCTGCAGCCACCTGATC | GTTCCGAGCCGCTTCCTTGGCTGCC |
| Sox17 | GATGCGGGATACGCCAGTG | CCACCACCTCGCCTTTCAC |
| T (Brachyury) | TTGGGTAGGGAGTCAAGACTCCTGG | AGCTCGCTCTCCTCCAGGCCCACTC |
| 1600029D21Rik | TCCGACATCTTGGAAATCAGC | GGGACCGTCACTGTATAGGTTACA |
| 1190005I06Rik | TGAGCGTGCCCAACATTATC | GACTTGCTTGTTAGAATCCCGG |
| Bcam | CCTCACCCTGCACTATCCCA | CCGTGGTGGACGGGC |
| BMP4 | ATGAGACACCATGATTCCTGGTAA | GCGCCTCCTAGCAGGACTT |
| Brunol4 | GGGTGCGCCTTTGTGAAGTA | GCTCCAGGCATGGTCTGG |
| Cd9 | CTTGTACCATGCCGGTCAAA | CAGCGAGCCAGAAGATGAAGT |
| Cyp2s1 | TTTGCTGGGACCATGACCAT | TCCCGGACGCGCTG |
| Depdc6 | ACCATGCCACATCCAGGCT | GACGACAAACTGACAGACCTTCAT |
| Dgka | CGTTGAGATATGCGGGAAGC | TCAATACCGCAATGCCTTCTAG |
| E130012A19Rik | CTCCGCCAGGAACATTTCAA | TGGAGTGTCCAGGGCGAA |
| Efemp1 | AACCCTTCCCACCGGATC | ATCTTGGCACACATTATGCTCACT |
| Epha2 | GCTACGGCATTGTCATGTGG | CTTTCATGACCTCGTGGTTTGA |
| Fgf17 | AGTTCGCCAAGCTCATCGTG | TCTCTGCCCCCTTGATGC |
| Grtp1 | CCTACCTGTGGAGACGGTGC | GAGCAACCCGGAATATGATTTT |
| Hck | TGGATGGCCACCTACAACAA | CAGGAAGGCCTCCACGG |
| Igfbp3 | AGACAGAATACGGTCCCTGCC | ACATTGAGGAACTTCAGATGATTCAGT |
| Lamc2 | TATCGAAGGTTACTGCGGAACC | CGTTATCAATGTACCCTGTACTATATTCTCC |
| Ltbp4 | TGAGCGTGGAAGCTATACAGGA | GCAGCCATCCAGGATACCA |
| Mras | TGGATGTTCTGGACACAGCC | AAGCCATCCCCTGTGCG |
| Nedd4l | TTACTACAGTTTGTCACAGGCACCT | TGAGGACCATTGGAACCATAGAG |
| Niban | GTTTCCATGAGGCCGCC | TCATGTAATCGTGGTTCAGGTGTC |
| Ntn1 | GAGGCCAACGAGTGCGTG | GCTTATAGAGCTCCATGTTGAATCTG |
| Perp | CAGAGCCTCATGGAGTACGCA | CACAGGATGATAAAGCCACAGAAA |
| Serpinb6c | AGAAGGATACTGGCTCACCATGA | AGGTTTAAGGCAAAAGTAGCATTTG |
| Serpinb9b | GAATCTGGAAGGCTGCTGTGT | AGAGTCTGAGTAGCTGCTGCTCAA |
| SFN | CTGCACACCCTCAGTGAGGA | TGTCCACAGCGTCAGGTTGT |
| Sirt4 | GAATAAGAATGAGCGGATTGACT | CGGCTGAGGTGGGTGATC |
| Tcl1 | GGCACACAGGGCAGAGACACCTGCA | CAGGATCTGCCAATACATGGAGTCG |
| Trim2 | GTGTGGCCGTGGACTCAAAT | CCTGGATCCTGCTGTTCCC |
| Ankrd1 | GAAGGACACTTGGCGATCGT | TGGATTCAAGCATATCTCGGAA |
| Cadm1 | CTGCCAGCTCTACACGGACC | AGTTACGTGGAGGAACCAGGAC |
| Cald1 | CGTCCGCAATATCAAGAGCAT | TCCTTATTGGGTGTTCCTGAGG |
| Cdc42ep1 | ACCAGGCCACCTATGACAGTCT | GTAACCAGAGTGGCCATCTGTG |
| Cgln1 | AGCACTCAGGCCACACCAG | TTCGTTTGTTGTGTGAGCTCCT |
| Cnn2 | CGAGGTCAAGAACCGGCTC | TCGGAGTTCTGCTTCCTTTTG |
| Cotl1 | ATCCAGCAGTGCACAGATGATG | CTTGGATCTTTTGCTCATGGC |
| Cttnbp2nl | CCGCAAGGTGATCCTCGA | GCATGTAGGTGACATCATCTCCTTC |
| Dtna | TCAAGCCGTGAACCTTTGC | TCTCGGAGGCACGATGTGA |
| Ets1 | TGCCATCAAGCAAGAGGTGTTA | CCCCGAGTTTACCACGACTG |
| Igfbp7 | CTGCGAGCAAGGTCCTTCC | TCACAGCTCAAGAACACCTTGG |
| Itpr1 | CCCAGCGGCTGCTAACC | TGGAAAGCCTGACCCAGGT |
| Lrrk1 | TCACAGGAGAAGGGCCAGC | CGCTTCTCAGTGAGCAGGAACT |
| Ltbp1 | CCCCGTCCAGGGAACTG | GTGTCGTAAGAGGATTCTCCAGC |
| Mfhas1 | ACCCTAGAGAAGCATCGTAGATGAG | ACCCTAGAGAAGCATCGTAGATGAG |
| Mmp2 | GACAAGAACCAGATCACATACAGGAT | AGGCCCGAGCAAAAGCAT |
| Mtap1b | AAGTGACGTCTTAGAAACAGTCGTTC | GATCATCAAACGCACCTCAGTG |
| Mtap7d1 | TCAAGAAGCGCCAGTCGC | TTGGACTTAGGGCTGAGCTCA |
| Plekhg2 | AACAGCGCAGGCAGCC | CACAGTGCTCAACGAAGTGGAG |
| Rgnef | GCCCTGAGAGAAGCCGAGA | TCGGATGACTGGCTTACATCG |
| Runx1 | TGCTCCGTGCTACCCACTC | CCCCAGTGCCACCACCTTGAA |
| Sall2 | CCCCAACAGTTAATCTCGGACT | TTCCTCGCTAGCATCACCGT |
| Serpine1 | CTACACTGAGTTCACCACCCCC | TGAACATGCTGAGGGTGTCG |
| Shroom3 | CGGTCCCTGTGCATGTGA | TCCCTCAGGAGCACATCCTG |
| Slc22a23 | CATTGCTGACTGGGTTGGC | CACAGTCAGTCCAAAGATGAGGAT |
| Specc1 | CGAGTCTCGGCTGAGTAAGCT | TCTTCAGTCTCGCCACCCC |
| St6gal1 | AGCTGGGTCGAGAGATTGATAATC | TCTGTAGGTGCCCCATTAAACC |
| Syt11 | GACAGTGGTGGTCCTCAAAGC | TTCACCTTGACATAAGGATTACCTGA |
| Tgfβ2 | CCTTCGTGCCGTCTAATAATTACA | CCATCAATACCTGCAAATCTCG |
| Tmem2 | AGGTTTCGGCCCCATCAG | CTGTCGATGATGGCTGCAAC |
| Tshz1 | GGCGCTCGGCAGCTTAC | CCATCCTCCGCGTGCTC |
| Gapdh | GTATGACTCCACTCACGGCAAA | TTCCCATTCTCGGCCTTG |
