# Supplementary material for: Direct targets of Klf5 transcription factor contribute to the maintenance of mouse embryonic stem cell undifferentiated state
Source: BMC Biol. 2010 Sep 27;8:128. doi: 10.1186/1741-7007-8-128 (PMC2955566; doi:10.1186/1741-7007-8-128)
Supplement: Additional file 13 — Additional Table 6. Primers used for ChIP-qPCR. [file 1741-7007-8-128-S13.DOC]

**Additional Table 6:** Primers used for ChIP-qPCR.

| **Sample*** | **Forward primer** | **Reverse primer** |
| --- | --- | --- |
| 1 | GAGGCGCGGGAACCA | TTGTTATTGCGGAGCAGATGTC |
| 2 | TCCCTAGCTGCGTTCTTTGC | AAGGTTGCAGATGCCAGTGAC |
| 3 | AGGGTAAGCAGGGAGTCTGTAGTG | CCTCTCTGCCCCCACACA |
| 4 | GCCATCCCTCAGCCTCAGA | CGCAAGTTCTCCAGCTGTTG |
| 5 | GAGCCTTTTACTGGGTGCTGA | ACTTTCCCACATGAAGTACAGGG |
| 6 | CAGCTGCCTGCTCATTTGC | TTCACCTGGGCAGTATTTAGATTG |
| 7 | CAAAGACAAAAACCACATGATCATC | GAATGGGTGTTGGATTTTGTCA |
| 8 | AACAGTTAAGTGGTCAGCAGAAAGG | GTAGCATGGCAGGGCAGC |
| 9 | CTGGCACGAGAAGCAGGG | TTGGCTGGTCGCCACTTT |
| 10 | GGCCAGGGTTGTGGAGC | CACTGGCTTTGGTCACCTAGACT |
| 11 | CCCAAGACTCTCACCATTGACTT | GCTATGCTGCAGATGACCACAG |
| 12 | ACTCACCGCAAAGCAGCC | AGCCGCTTTGAAAACTACGG |
| 13 | GGGAACTCTACCCAACTCATTCTATG | GGTGGATCTTTCTGTGGTTTAGTTATC |
| 14 | GGGCAGCTCTGCTCCTCTT | CAGTCTTCCTTTTCACCAGTCTATGT |
| 15 | AGCCTGATGGCGACTTTTGT | CTCTCTGGCTGCGTGTGAAG |
| 16 | CTCAAGTGCTCTGAGATTCAGCA | GGGCACTGAGCAACAGTGAATAC |
| 17 | CCTATTGTGGGTGGGAACCA | TGGTTTGCTCAGCCTACTTTCTTA |
| 18 | TCCTGGCCCCTGAATGG | TTGCGGGACAAGGAACATC |

***** See **Additional Figure 2**
